# Erucic acid concentration of rapeseed (*Brassica napus* L.) oils on the German food retail market

**DOI:** 10.1002/fsn3.2327

**Published:** 2021-05-11

**Authors:** Marco Russo, Feng Yan, Annegret Stier, Linda Klasen, Bernd Honermeier

**Affiliations:** ^1^ Institute of Agronomy & Plant Breeding I Justus Liebig University Giessen Giessen Germany; ^2^ Nutritional Epidemiology Department of Nutrition and Food Sciences University of Bonn Bonn Germany

**Keywords:** *Brassica napus* L., erucic acid, fatty acids, food safety, infant nutrition, rapeseed oil

## Abstract

Rapeseed oil is one of the most important vegetable oils in Germany. It has a favorable fatty acid composition but also contains a certain amount of erucic acid (EA). As the result of toxicological considerations regarding this fatty acid, the European Food Safety Authority (EFSA) established a tolerable daily intake (TDI) for EA of 7 mg/kg body weight in 2016. On this basis, the maximum EA levels for vegetable oils allowed in the European Union have been reduced shortly from 50 to 20 g/kg, and for infant formula and follow‐on formula from 10 to 4 g/kg. However, rapeseed oil is also recommended for the preparation of homemade food for infants and children. Little is known about the actual EA concentrations of rapeseed oils on the German retail market. Current data are especially important for the necessary reassessment of its recommendation in infant and child nutrition based on the established TDI. Three hundred representative rapeseed oil samples were purchased in retail stores across Germany. EA concentrations, determined by GC‐FID, were in a range of 0.17–9.68 g/kg, with 241 samples being even below 4 g/kg. All oils were below the maximum level valid at the time of this investigation, and even below the newly established lower maximum level of 20 g/kg. The major part also met the requirements for infant and follow‐on formula. The representative results provide valuable current data for the necessary reassessment of the dietary recommendations for infant and child nutrition based on the established TDI.

## Graphical abstract

The recommendation of using rapeseed oil for infant and child nutrition needs to be reassessed due to its erucic acid content and based on a tolerable daily intake (TDI) established by EFSA. Thus, this study investigated a large and diverse dataset of rapeseed oil samples found on the German retail market, where 241 out of 300 rapeseed oils exhibited erucic acid concentrations below 4 g/kg, the maximum level for infant and follow‐on formula. For the necessary reassessment of the dietary recommendations for infant and child food based on the EFSA TDI, our investigation provides valuable data.

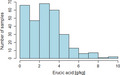

## 1 INTRODUCTION

In the first half of the 20th century, rapeseed oil was of little acceptance and importance as an edible oil because of its unfavorable sensory properties and the high concentration of erucic acid, a fatty acid regarded as nutritionally and toxicologically negative. Only with the breeding of rapeseed varieties with low levels of erucic acid and glucosinolates, referred to as “double low” or “double zero (00)” varieties (later often labeled as canola), and the advances in production and processing technology, the conditions for using rapeseed oil as an edible oil improved significantly. Today, rapeseed oil is one of the most important vegetable oils in Germany (UFOP, 2018), and number three worldwide after palm and soybean oil (USDA, 2020). It is regarded as a nutritionally valuable edible oil (Kruse et al., 2015; Lin et al., 2013) due to its favorable fatty acid composition (Deutsche Lebensmittelbuch‐Kommission, 2011), and therefore also recommended to be used in infant and child nutrition (Hilbig et al., 2012; Stimming et al., 2015).

**FIGURE 1:**
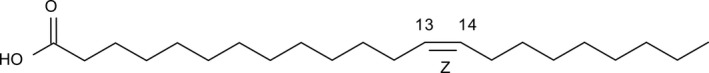
Chemical structure of erucic acid (*cis*‐13‐docosenoic acid), with the double bond at position 13 in *cis*‐configuration

Erucic acid is a mono‐unsaturated fatty acid with 22 carbon atoms, also known as *cis*‐13‐docosenoic acid (22:1Δ13*c*) due to the double bond at position C‐13 (Figure 1). Counting from the methyl group of the molecule, the double bond is positioned at n‐9 (or ω‐9), thus classifying erucic acid as an omega‐9 fatty acid. It can be found in the seeds of many species within the Brassicaceae family, like rapeseed (*Brassica napus*), or black (*Brassica nigra*), brown (*Brassica juncea*), and white mustard (*Sinapis alba*), whereas its concentration is only negligible in the plant parts used as cruciferous vegetables, like broccoli, red cabbage or white radish (Vetter et al., 2020).

Rapeseed oils rich in erucic acid (HEAR = high erucic acid rapeseed) are still used for technical purposes. In contrast, only rapeseed oils with very low levels of erucic acid (LEAR = low erucic acid rapeseed), which are considered toxicologically safe, are permitted for human consumption. With regard to human erucic acid intake, the EFSA CONTAM panel established a TDI (Tolerable Daily Intake) of 7 mg/kg body weight (Knutsen et al., 2016). This value was determined from data obtained from animal models, taking into account an uncertainty factor for transmission to the human organism. Based on these considerations and in accordance with the Codex Alimentarius (Codex Alimentarius Commission, 1999), the maximum erucic acid concentration of 50 g/kg (European Commission. Commission Regulation EU, 2014) for vegetable oils has been reduced in the European Union to 20 g/kg in 2019 by EU regulation 2019/1870 (European Commission. Commission Regulation EU, 2019a). Since infants are generally considered a particularly vulnerable group, lower maximum levels are set for infant formula and follow‐on formula. Accordingly, these products were allowed to contain erucic acid concentrations of up to 10 g/kg in the fat phase (European Commission. Commission Regulation EU, 2014), while this value was reduced to 4 g/kg in 2019 (European Commission. Commission Regulation EU, 2019b). However, rapeseed oil is also recommended to be used in the preparation of homemade food for infants and children (Hilbig et al., 2012; Stimming et al., 2015). It remains unclear whether the rapeseed oils sold on the retail market could lead to erucic acid concentrations in the homemade food exceeding the TDI established by EFSA.

In order to be able to validly assess the intake of erucic acid in human nutrition, especially in infant and child nutrition, current data on the erucic acid concentration in rapeseed oils sold on the retail market are required. Since these were not available to date, the current project aimed at examining a large, diverse and representative set of rapeseed oils offered in German food retailing. In particular, this should create a data basis for the reassessment of the recommendations for the use of rapeseed oils in infant and child nutrition that are necessary based on the EFSA TDI.

## 2 MATERIAL AND METHODS

### 2.1 Rapeseed oil sampling

Rapeseed oil samples were purchased from food retailers in Germany. The aim was to conduct the sampling roughly based on the market share of the rapeseed oil brands sold in German food retail. Since around 90% of the German rapeseed oil market is covered by only six food retail chains (Nielsen, 2018), these were selected for sampling. Rapeseed oils with a market share of at least 0.05% were taken into account.

In order to adequately consider rapeseed oils with a relatively low market share, and at the same time to avoid buying too many samples of the same brand with a high market share, the final sample plan showed a certain degree of deviation from the actual market share. This ensured a broader coverage of the rapeseed oil spectrum available in German food retail stores.

To consider possible geographical differences within Germany as well as possible differences between urban and rural areas, the shopping locations were divided into four geographical regions (East, North‐West, West, South; see Table 1). Within each region, a large city, a medium‐sized city, and a rural area were selected. This procedure was based on the BfR MEAL study (Lindtner & Sarvan, 2019).

**TABLE 1:** Regional breakdown of the sampling of rapeseed oil in German food retail stores (sum of both sampling periods)

Region	Location	Samples	Region	Location	Samples
1 (East)	Berlin^a^	35	3 (West)	Cologne^a^	35
	Gera^b^	25		Worms^b^	27
	Crivitz^c^	15		Kirtorf^c^	13
2 (North‐West)	Hamburg^a^	33	4 (South)	Munich^a^	35
	Celle^b^	28		Aalen^b^	25
	Augustfehn^c^	14		Nittenau^c^	15

Note

At the places in the rural areas, some samples were also bought in neighboring small towns or villages.

^a^ Large cities (>500,000 inhabitants).

^b^ Medium‐sized cities (50,000–100,000 inhabitants).

^c^ Rural areas (<10,000 inhabitants).

A total of 300 rapeseed oil samples (including four samples specifically designated for the preparation of complementary foods for infants) were purchased from German food retailers, with 75 samples in each of the four geographic regions (see Table 1). Broken down by location size, this resulted in a number of 138 samples in large cities, 105 samples in medium‐sized cities, and 57 samples in rural areas.

To take into account a possible variation in the quality of the rapeseed oil samples over time, purchasing of the samples took place in two periods with 150 samples each (1st sampling: 10 April–06 May 2019; 2nd sampling: 27 June–20 July 2019).

During the purchasing, some deviations from the original sampling plan were inevitable, since not all rapeseed oils were sold in the intended stores/regions, or were sometimes not at all available in the food retail stores. In these cases, comparable oil samples were purchased.

When purchasing the samples, the name of the rapeseed oil, the date of purchase, the place of purchase including the location, the best before date, the fill volume of the container, the bottle material, and, if available, the batch number were documented. In addition to the 300 rapeseed oil samples, 12 additional oil samples specifically designated for the preparation of complementary foods for infants were purchased, irrespective of the sampling periods mentioned above. However, most of these (i.e., eight samples) were not pure rapeseed oils, but rather mixtures with other vegetable oils. Therefore, these additional oil samples were excluded from the evaluation in order not to distort the results.

After purchase, the oil samples (closed bottles) were stored in a cold room at 5°C in the dark. The bottles were only opened at the beginning of the laboratory analysis. The number of samples, characterized by quality (refined/cold‐pressed, rapeseed oil/ rapeseed kernel oil, organic/conventional), is shown in Table 2.

**TABLE 2:** Erucic acid concentration [g/kg] of rapeseed oil samples from the German retail market 2019. Results of descriptive statistics for the whole dataset, as well as divided by categories

Categories	*n*	Min	*Q*_1_	Median	Mean	*Q*_3_	Max	*SD*
Total	300	0.17	1.26	2.67	2.71	3.51	9.68	1.78
Sampling period								
1	150	0.33	1.25	2.50	2.69	3.59	9.10	1.84
2	150	0.17	1.38	2.76	2.72	3.45	9.68	1.72
Sampling region								
1	75	0.17	1.30	2.41	2.52	3.32	9.68	1.83
2	75	0.17	1.82	2.89	2.86	3.59	8.77	1.71
3	75	0.43	1.45	2.80	2.85	3.54	7.86	1.81
4	75	0.31	1.02	2.43	2.60	3.49	7.11	1.77
Extraction method								
Cold pressed	91	0.31	0.92	1.78^a^	2.39	3.62	8.77	1.76
Refined	209	0.17	1.75	2.78^b^	2.84	3.49	9.68	1.78
Raw material								
Kernel oil	47	0.62	0.94	2.04	2.34	3.43	7.29	1.58
Seed oil	253	0.17	1.45	2.69	2.77	3.50	9.68	1.81
Production method								
Organic	32	0.33	0.75	0.97^a^	1.33	1.47	3.65	0.91
Conventional	268	0.17	1.73	2.78^b^	2.87	3.63	9.68	1.79

Note

Different lower case letters indicate statistically significant group differences within the category (Mann‐Whitney test). See text for further explanation.

Abbreviations: *n*, number of samples; *Q*_1_, first quartile; *Q*_3_, third quartile; *SD*, standard deviation.

### 2.2 Chemicals and standards

Chloroform was obtained from Merck. Isooctane, potassium hydroxide, methanol, sodium chloride, and fatty acid methyl ester mixture ROTICHROM® ME 51 were purchased from Carl Roth. Anhydrous sodium hydrogen sulfate (NaHSO_4_) was obtained from Bernd Kraft. The internal standard triheneicosanoin was purchased from Larodan AB, Sweden. Fatty acid methyl ester mixture Supelco 37 Component FAME Mix (certified reference material) was obtained from Sigma Aldrich.

### 2.3 Transesterification of rapeseed oil samples

Erucic acid concentration of the rapeseed oil was determined according to DIN EN ISO 12966‐2:2017‐08 (DIN EN ISO 12966‐2, 2017), 5.2 (“Rapid method” under alkaline conditions), additionally using triheneicosanoin (triacylglycerol of heneicosanoic acid, C21:0) as an internal standard (IS).

Internal standard was solubilized at 1 mg/ml in chloroform. One milliliter of IS solution was pipetted into test tubes and evaporated until dryness. After the addition of about 75 mg of the rapeseed oil sample and 2 ml of isooctane, test tubes were closed and shaken vigorously. For full dissolution of the IS, the test tubes were placed in a warm water bath at 50°C for 10 min. After the addition of 100 µl of a methanolic potassium hydroxide solution (2 mol/L), test tubes were again closed and shaken vigorously for 1 min. After allowing the test tubes to stand for 2 min, 2 ml of a saturated NaCl solution (40 g NaCl in 100 ml distilled water) was added, shortly shaken, and left to stand until clear phase separation was achieved. The upper phase was transferred to a new test tube, and after the addition of about 1 g of anhydrous sodium hydrogen sulfate and a shaking step, the clear solution was transferred into GC vials. All samples were prepared in duplicate.

### 2.4 Determination of erucic acid concentration by GC‐FID

Determination of fatty acid methyl esters (erucic acid and heneicosanoic acid as IS) was performed with a GC system CHROMPACK CP‐3800, with autosampler 8200 CX, flame ionization detector (FID), and the software Varian Star 5.3 (all from Varian). Separation of the fatty acid methyl esters was performed on a Stabilwax‐DA GC column from Restek (30 m, 0.32 mm ID, 0.25 µm). Helium was used as a carrier gas with a flow rate of 1 ml/min. The split ratio was set to 1:20. The injector temperature was 280°C.

The temperature program (total run time 29 min) was as follows: 160°C [1 min] ‐> [10°C/min] ‐> 220°C [8 min] ‐> [10°C/min] ‐> 240°C [12 min]. Consistency of the retention times within a measuring sequence was controlled by injection of fatty acid methyl ester mixture ROTICHROM^®^ ME 51. For quantitative evaluation, the GC factor was determined by six‐fold measurement of fatty acid methyl ester mixture Supelco 37 Component FAME Mix (certified reference material), and calculation as a ratio of the measured peak areas and the certified values according to the following formula:

$$\text{GC}\;\text{factor}=\frac{{\text{mass}}_{\text{erucic}\;\text{acid}}}{{\text{mass}}_{\text{IS}}}\times \frac{\text{peak}\;{\text{area}}_{\text{IS}}}{\text{peak}\;{\text{area}}_{\text{erucic}\;\text{acid}}}$$

Calculation of the erucic acid concentration, expressed as g/kg rapeseed oil, was performed according to the following formula:

$$\text{Concentration}\left[\frac{g}{\text{kg}}\right]=\frac{\text{peak}\;{\text{area}}_{\text{erucic}\;\text{acid}}}{\text{peak}\;{\text{area}}_{\text{IS}}}\times \frac{{\text{mass}}_{\text{IS}}}{{\text{mass}}_{\text{oil}\;\text{sample}}}\times 1000\times \text{GC}\;\text{factor}$$

### 2.5 Statistical analysis of laboratory analyses

Statistical analysis was performed with R (Version 3.6.1) (R Core Team, 2019). Due to deviations from the normal distribution, the nonparametric Mann–Whitney test (two groups) and Kruskal–Wallis test (four groups) were used, with a significance level of *α* = 5%.

## 3 RESULTS

The measured erucic acid concentrations of the investigated samples ranged from a minimum of 0.17 to a maximum of 9.68 g/kg (Table 2). All of the examined rapeseed oil samples (Table S1)thus met the legal requirements of a maximum level of 50 g/kg for vegetable oils with regard to EU Regulation 696/2014 (European Commission. Commission Regulation, 2014), which was valid at the time of this investigation, as well as the new maximum level of 20 g/kg, as established at the end of 2019 by EU regulation 2019/1870 (European Commission. Commission Regulation, 2019a).

**FIGURE 2:**
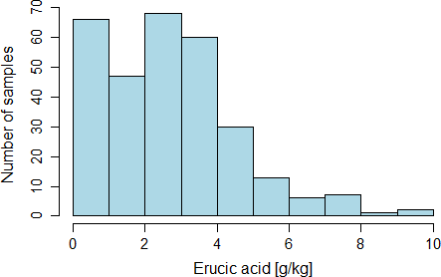
Histogram of erucic acid concentrations of the 300 investigated rapeseed oil samples purchased on the German retail market in 2019

Erucic acid concentrations of the investigated rapeseed oil samples were not following a normal distribution, but rather showing a right‐skewed, two‐peak distribution (cf. Figure 2). This shows that samples with low erucic acid concentrations clearly predominated within the determined range. Two hundred seventy‐one samples had erucic acid concentrations below 5 g/kg, and 241 samples were even below 4 g/kg.

The key figures from the descriptive statistics of the investigated rapeseed oil samples are shown in Table 2. Neither the two sampling periods nor the four geographic regions showed significant differences regarding the erucic acid concentrations. Therefore, the rapeseed oils from both sampling periods and all regions were pooled and evaluated together in the subsequent evaluations.

**FIGURE 3:**
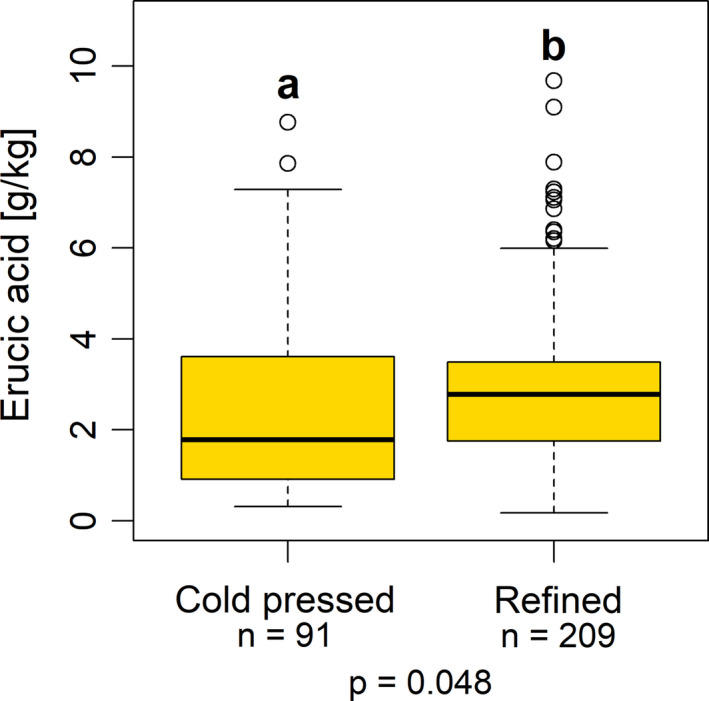
Erucic acid concentrations of the investigated rapeseed oil samples (*n* = 300), cold‐pressed versus refined oil. Different lower case letters indicate statistical significant group differences (*p* = .048; Mann‐Whitney test)

Erucic acid concentrations of the cold‐pressed oils were ranging from 0.31 to 8.77 g/kg, with a median value of 1.78 g/kg. For the refined oils, a range from 0.17 to 9.68 g/kg was found, with a median value of 2.78 g/kg (Table 2). As evaluated by Mann–Whitney test, these median values differed significantly from each other (*p* = .048; Figure 3).

**FIGURE 4:**
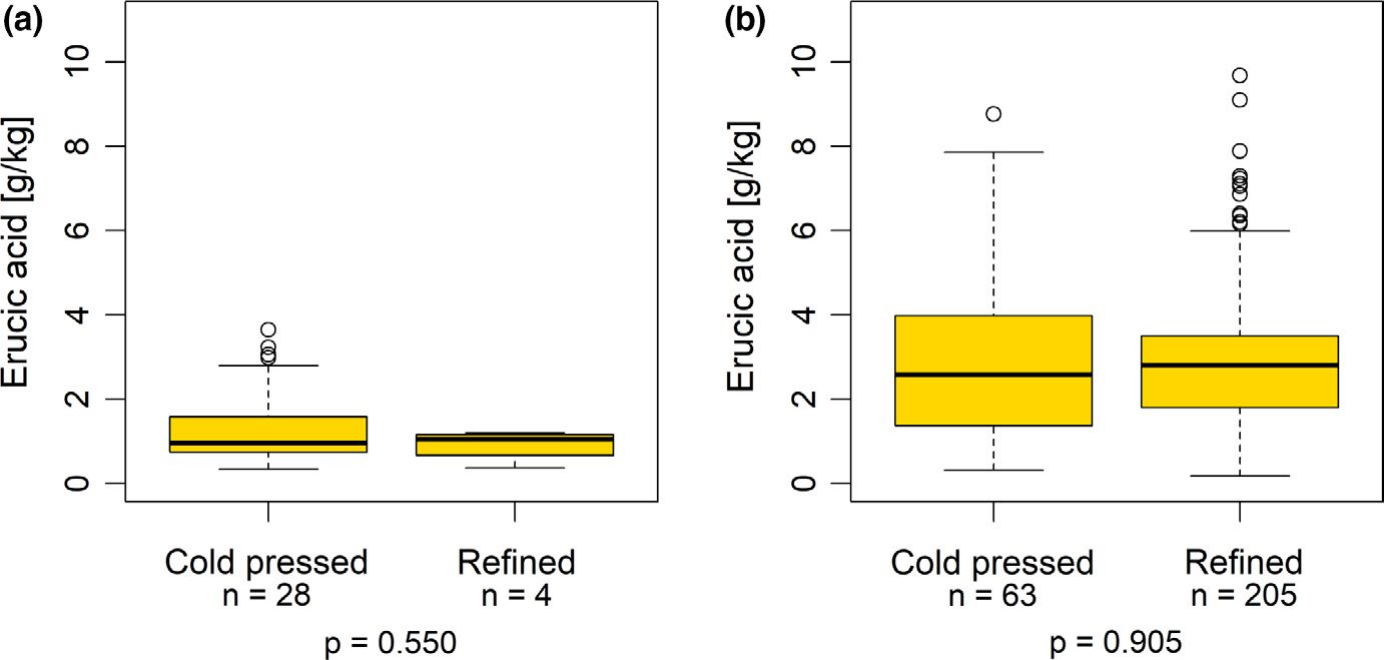
Erucic acid concentrations of cold‐pressed and refined oil samples within the subgroups of organically and conventionally produced oils: (a) Organically produced oils (*n* = 32; *p* = .550; Mann‐Whitney test); (b) Conventionally produced oils (*n* = 268; *p* = .905; Mann‐Whitney test)

However, within the subgroups of organically and conventionally produced rapeseed oils, no such difference between cold‐pressed and refined oils were found (Figure 4).

**FIGURE 5:**
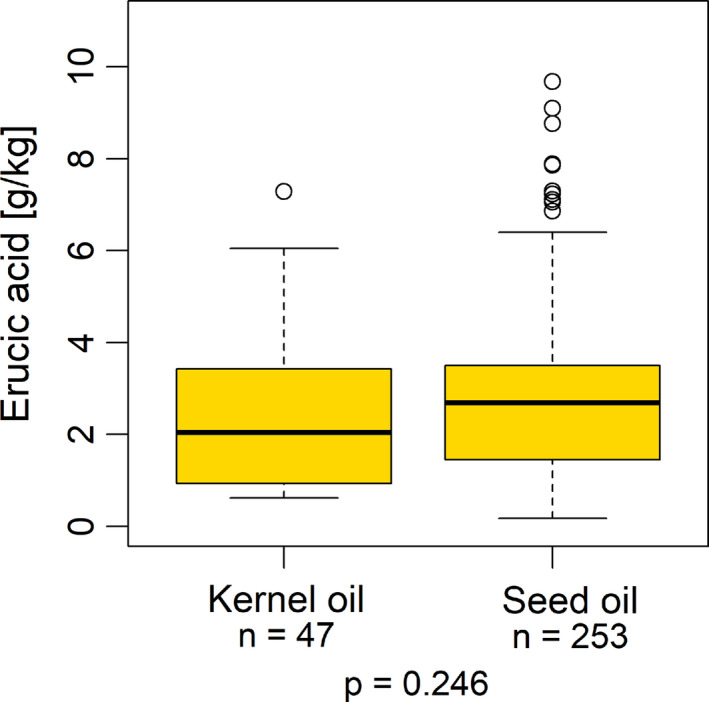
Erucic acid concentrations of the investigated rapeseed oil samples (*n* = 300), rapeseed kernel oil versus rapeseed oil. No statistically significant group differences (*p* = .246; Mann‐Whitney test)

Rapeseed kernel oil samples covered a range from 0.62 to 7.29 g/kg, with a median value of 2.04 g/kg. Erucic acid concentrations of rapeseed oil samples processed from whole seeds were in a range from 0.17 to 9.68 g/kg, with a median value of 2.69 g/kg (Table 2). However, as calculated by Mann–Whitney test, these two groups of oil samples did not differ significantly from each other (*p* = .246; Figure 5).

The range of erucic acid concentrations found in the 32 examined organically produced rapeseed oil samples was from 0.33 to 3.65 g/kg (median value 0.97 g/kg), whereas the 268 conventionally produced rapeseed oil samples exhibited a range from 0.17 to 9.68 g/kg (median value 2.78 g/kg; Table 2). Although a comparison of the samples with the Mann‐Whitney test showed a significant difference between the two groups (*p* < .001), it has to be kept in mind that the sample sizes of the two groups were very different (*n* = 32 vs. *n* = 268), limiting the ability to derive general statements.

Among the 300 rapeseed oil samples, four were specifically designated for the preparation of complementary foods for infants. These were all organically produced, refined oils from the same manufacturer, and exhibited very low erucic acid concentrations, covering a range from 0.37 to 1.19 g/kg.

## 4 DISCUSSION

### 4.1 Variation of erucic acid concentration in rapeseed oil samples and legal requirements

All of the investigated rapeseed oils met the legal requirements regarding maximum erucic acid levels of 50 g/kg, as prescribed in EU regulation 696/2014 (European Commission. Commission Regulation, 2014), which was valid at the time of this investigation. Even according to the new specification of EU regulation 2019/1870 (European Commission. Commission Regulation, 2019a), which established a lower maximum level of 20 g/kg, the investigated rapeseed oils met these stricter requirements.

Oils with rather low concentrations of erucic acid predominated among the 300 investigated samples, which showed erucic acid concentrations ranging from 0.17 to 9.68 g/kg. These values were on average lower than those reported by Matthäus and Brühl (2003), who investigated 48 cold‐pressed and two refined rapeseed oils in Germany more than 15 years prior to our investigation. In their investigation, a range from 0.4 to 21.7 g/kg was found, with an average of 3.7 g/kg. However, only two oils were found with erucic acid concentrations above 10 g/kg (Matthäus & Brühl, 2003), thus showing a right‐skewed pattern similar to the pattern found in our investigation regarding the distribution of erucic acid concentrations among the investigated oil samples.

In our investigation, approximately 90% of the samples (90.3%; 271 samples) had erucic acid concentrations below 5 g/kg. Around 80% of the samples (80.3%; 241 samples) were even below 4 g/kg, and thus below the maximum erucic acid concentration for infant and follow‐on formula which was only quite recently changed (during the execution of our investigation) from 1% to 0.4% in 2019 (European Commission. Commission Regulation, 2019b). In the preparation of homemade infant and child foods, these oils would thus not lead to erucic acid concentrations exceeding the concentrations allowed for infant and follow‐on formula. The four samples that were specifically designated for the preparation of complementary foods for infants were well below this limit.

Already in the 1960s, the erucic acid concentration in rapeseed oil was reduced up to <2% due to the work of Canadian plant breeders (Stefansson & Hougen, 1964). This success was based on the elucidation of the genetic background regarding the synthesis of erucic acid and its inheritance in rapeseed (Downey & Craig, 1964; Harvey & Downey, 1964). In contrast to Canada, almost only winter rapeseed is grown in Central Europe, with which the selection work is more time‐consuming than with summer rapeseed. Thus, LEAR varieties were only introduced in Germany in the early 1970s. Since then, only rapeseed oils with a low erucic acid concentration have been available on the German food market.

The high proportion of rapeseed oils with very low erucic acid concentrations reflects the successful breeding of LEAR varieties. It also illustrates that the rapeseed oil manufacturers exercise the necessary care when selecting their raw materials, as already a small percentage of HEAR within a batch of LEAR can cause dramatic changes of the erucic acid concentration of the whole batch (Vetter et al., 2020).

### 4.2 Variation of erucic acid concentration in rapeseed oil sample subgroups

The finding that the erucic acid levels did not differ significantly between the two sampling periods examined appears reasonable due to the relatively short interval between the two sampling periods. Thus, it can be assumed that the manufacturers used comparable batches of raw materials from the same harvest year for rapeseed oil production. In order to record possible annual fluctuations in the erucic acid concentration between different harvest years, investigations over several years would be necessary.

Also between the four regions examined, erucic acid levels did not differ significantly. This can be attributed to the small number of large oil mills in Germany that supply larger areas, as well as the centralized purchasing of the large food retail chains. Only a few regionally produced oils can be found on the market. In addition, raw materials are purchased nationwide and even internationally by the larger oil mills. Therefore, especially oils from regionally produced raw materials are hard to be expected. Nevertheless, the pre‐treatment of rapeseed and the method by which rapeseed oil is extracted from the press cake can influence its quality (Guo et al., 2019; Yu et al., 2020; Zhou et al., 2019). There were indications that the pre‐treatment of rapeseed by steam explosion led to a better oil quality as compared to the traditional high‐temperature roasting (Yu et al., 2020). In addition, it was shown that the use of butane as an extraction agent increased the extracted oil yield and the concentrations of ß‐carotene, tocopherol, and canolol in the oil and slightly reduced the erucic acid concentrations (Guo et al., 2019).

Refined rapeseed oils differ from cold‐pressed oils in various processing steps carried out after pressing. These serve to remove ingredients classified as undesirable, like constituents that negatively affect the taste, smell, or shelf life, but also substances that are harmful to health, such as pesticide residues or heavy metals. The latter is one of the reasons why refined oil is often used in infant nutrition. However, refining also reduces desirable substances such as fat‐soluble vitamins. To what extent refining could influence the fatty acid composition appears unclear. Possibly, it could be modified slightly by minor deviations in the fatty acid patterns between triacylglycerols, the main constituent of an oil, and for example, phospholipids, which are removed during refining in the course of the so‐called degumming. Rapeseed phospholipids contain only small amounts of erucic acid (Persmark, 1968; Sosada et al., 1992), and have a lower proportion of erucic acid than triacylglycerols (Zaderimowski & Sosulski, 1978). Thus, a shift in the erucic acid concentration after degumming appears conceivable. However, since the proportion of phospholipids in pressed rapeseed oil is relatively low (Eskin & Przybylski, 2003; Zaderimowski & Sosulski, 1978), it is more likely that the results of the current investigation are related to the quality of the raw material than to a processing effect.

Regarding the raw material, it might be conceivable that a connection exists between the extraction method and the production method, that is, that the cold‐pressed oils could mainly be extracted from organically produced rapeseeds, and the refined ones mainly from conventionally produced rapeseeds. In such a case, the differences between the extraction methods could at least partially be explained by the different raw materials from the two production methods (see below). To a certain degree, this hypothesis can be supported by the findings of a closer examination of the structure within the subgroups. Among the 209 refined oils, only four (equaling 1.9%) were organically produced, whereas 205 (equaling 98.1%) were extracted from conventionally grown rapeseed. Although the organically produced oils did not dominate among the 91 cold‐pressed oils, their percentage (28 samples, equaling 30.8%) was much higher than in the group of refined oils. This shift in the proportions may at least partially explain the differences between cold‐pressed and refined oils in the whole dataset, which was not found within the subgroups, however. Therefore, it is likely that the different raw materials from the two production methods (as discussed below) have led to the results of the differences between the extraction methods. The question to what extent the extraction method itself has an influence on the erucic acid concentration of rapeseed oils would need to be specifically investigated in future studies by determining erucic acid concentrations of defined batches during the processing in an oil mill both before and after refining.

Rapeseed kernel oils differ from normal rapeseed oils in a dehulling step of the rapeseed grains before oil extraction (Rimbach et al., 2010). The seed coat (hull) contains waxes (Liu et al., 1996; O'Brien, 1998) and other fat‐soluble substances, which can affect the quality, taste, and aroma of the pressed rapeseed oil. Therefore, differences in the proportion and the composition of fat‐soluble substances would rather be expected than in the fatty acid spectrum and in particular in the erucic acid concentrations. However, the rapeseed hulls also contain a certain amount of oil, which can vary depending on the dehulling process (Carré et al., 2015, 2016; Koubaa et al., 2016; Mińkowski, 2002; Naczk et al., 1994; Yang et al., 2011). In our literature research, only statements on the oil concentration of the seed hulls (5.5%–21.2%), but no information on the fatty acid spectrum of the rapeseed hull oil was found. In addition, there are hardly any studies in the literature comparing rapeseed oil and rapeseed kernel oil obtained from the same raw material. In a study by Yang et al. (2011), only slight differences were found in the fatty acid composition of oils pressed from dehulled and whole rapeseed. Although the median of erucic acid concentrations of kernel oils was lower than the one of seed oils in our investigations, these differences were not significant. However, it would be an interesting approach to make targeted comparisons of rapeseed kernel oils and the associated rapeseed hull oils on several raw material batches in order to be able to derive statements on a possible different distribution of erucic acid in these two compartments.

Although our investigations found significant differences between organically and conventionally produced rapeseed oils, it needs to be emphasized that the number of organically produced samples was much lower than the number of conventionally produced oils, and that also conventionally produced samples with very low erucic acid concentrations were found. Since the number of organically and conventionally produced samples of the present study should be based on market shares, and the main question was not a comparison of these two production methods, the possibility to draw general conclusions is limited.

Differences between the two production methods are a strong restriction regarding the use of pesticides, as well as differences in the supply of nitrogen. The latter is generally lower in organic production, and based on the use of leguminous plants and organic fertilizers. With regard to lower nitrogen fertilization of LEAR varieties, results in literature state either no influence on the erucic acid concentration (Holmes & Bennett, 1979; Khan et al., 2018), or the erucic acid concentration was even slightly reduced by higher nitrogen fertilization (Zhang et al., 2012). It therefore seems unlikely that the lower erucic acid levels in the organically produced oils were caused by a lower nitrogen fertilization. In addition, selenium and sulfur also appear to have an influence on erucic acid concentration. Thus, fertilization with the two selenium forms selenite and selenate as well as with sulfur significantly reduced the erucic acid concentration of low erucic acid oilseed rape varieties (Davoudi et al., 2019; Liu et al., 2017; Shoja et al., 2018).

Possibly, an origin effect may play a role. Lower erucic acid concentrations were found at higher temperatures during the pod and seed formation period of the rapeseed plants (Wilmer et al., 1996; Yaniv et al., 1995), but higher erucic acid concentrations during drought stress (Bouchereau et al., 1996; Safavi et al., 2018; Ullah et al., 2012). Because organically produced rapeseed is often imported from other countries of origin, it is conceivable that the local climatic conditions could also have a possible impact on rapeseed oil quality. In addition, a genotype effect might play a role. Due to the restricted use of pesticides in organic agriculture, disease‐tolerant varieties are often preferred, even if they have lower yield potential, and these might also have a different potential of erucic acid production. As discussed before, the cultivars and their genetic background are the major factors influencing the erucic acid concentration. Thus, it is known that for erucic acid there is a high heritability, due to the high contribution of dominant regulators that control the biosynthetic pathway (Hatzig et al., 2018). In studies with different 00‐rape varieties or accessions containing <3% erucic acid, significant genetically determined differences in erucic acid concentrations were found. Depending on the genotypes used, this variation was in the range of 0%–3.3% (Davoudi et al., 2019; Hatzig et al., 2018; Safavi et al., 2018; Sharafi et al., 2015). It is, therefore, important for cultivation to know the erucic acid concentration of the varieties and to minimize it through further breeding.

Future investigations would need to be carried out with identical rapeseed varieties at the same location and under the same growing conditions to answer the question of whether the production process itself has an effect on the erucic acid concentration of rapeseed oils.

## 5 CONCLUSION

In conclusion, it can be noted that all the rapeseed oils purchased on the German market and investigated for the first time in a representative study met the requirements regarding erucic acid concentrations of edible oils for human consumption. They were well below the maximum level of 50 g/kg valid at the time of this investigation (European Commission. Commission Regulation, 2014), and even below the shortly established lower maximum level of 20 g/kg (European Commission. Commission Regulation, 2019a).

The major part also exhibited erucic acid concentrations below 4 g/kg, and thus even met the special requirements of the maximum level for infant and follow‐on formula which was only quite recently changed in the year of this investigation (European Commission. Commission Regulation, 2019b). These oils would thus not be disadvantageous for the preparation of homemade infant and child food compared to infant and follow‐on formula. For the necessary reassessment of the dietary recommendations for infant and child food based on the TDI established by EFSA, our investigation provides valuable data.

As the results are only based on 1 year, possible annual fluctuations are not covered. Thus, similar investigations should be performed regularly in the future to monitor the erucic acid concentrations of rapeseed oils on the German retail market.

## CONFLICT OF INTEREST

The authors declare that they have no conflicts of interests.

## Supporting information

Table S1

## Data Availability

The data that supports the findings of this study are available in the supplementary material of this article.
