# Supplementary material for: Erucic acid concentration of rapeseed (Brassica napus L.) oils on the German food retail market
Source: Food Sci Nutr. 2021 May 11;9(7):3664–72. doi: 10.1002/fsn3.2327 (PMC8269665; doi:10.1002/fsn3.2327)
Supplement: Supplementary file 1 — Table S1 [file FSN3-9-3664-s001.docx]

**Table S1.** Dataset of erucic acid concentrations of the investigated 300 rapeseed oil samples purchased on the German food retail market in 2019.
All samples were analyzed in duplicate.

| **Sample ID ^1^** | **Erucic acid [g/kg]** | **Sampling period ^2^** | **Sampling region** | **Location ^3^** | **Extraction method** | **Raw material** | **Production method** | **Complementary food oil ^4^** | **Location size** |
| --- | --- | --- | --- | --- | --- | --- | --- | --- | --- |
| 1 | 4.20 | 1 | North-West | Augustfehn | cold pressed | kernel oil | conventional | no | rural area |
| 2 | 1.89 | 1 | North-West | Augustfehn | refined | seed oil | conventional | no | rural area |
| 3 | 3.49 | 1 | North-West | Augustfehn | cold pressed | seed oil | conventional | no | rural area |
| 4 | 2.69 | 1 | North-West | Augustfehn | refined | seed oil | conventional | no | rural area |
| 5 | 3.64 | 1 | North-West | Augustfehn | cold pressed | kernel oil | conventional | no | rural area |
| 6 | 2.67 | 1 | North-West | Augustfehn | refined | seed oil | conventional | no | rural area |
| 7 | 2.49 | 1 | North-West | Augustfehn | cold pressed | seed oil | conventional | no | rural area |
| 8 | 4.03 | 1 | North-West | Augustfehn | refined | seed oil | conventional | no | rural area |
| 9 | 2.79 | 1 | North-West | Celle | refined | seed oil | conventional | no | medium-sized city |
| 10 | 3.50 | 1 | North-West | Celle | refined | seed oil | conventional | no | medium-sized city |
| 11 | 0.50 | 1 | North-West | Celle | refined | seed oil | conventional | no | medium-sized city |
| 12 | 2.67 | 1 | North-West | Celle | refined | seed oil | conventional | no | medium-sized city |
| 13 | 2.71 | 1 | North-West | Celle | cold pressed | kernel oil | conventional | no | medium-sized city |
| 14 | 4.06 | 1 | North-West | Celle | cold pressed | seed oil | conventional | no | medium-sized city |
| 15 | 3.59 | 1 | North-West | Celle | refined | seed oil | conventional | no | medium-sized city |
| 16 | 3.43 | 1 | North-West | Celle | refined | seed oil | conventional | no | medium-sized city |
| 17 | 4.24 | 1 | North-West | Celle | refined | seed oil | conventional | no | medium-sized city |
| 18 | 2.17 | 1 | North-West | Celle | cold pressed | kernel oil | conventional | no | medium-sized city |
| 19 | 0.45 | 1 | North-West | Celle | refined | seed oil | conventional | no | medium-sized city |
| 20 | 0.33 | 1 | North-West | Celle | cold pressed | seed oil | organic | no | medium-sized city |
| 21 | 0.49 | 1 | North-West | Celle | refined | seed oil | conventional | no | medium-sized city |
| 22 | 1.75 | 1 | North-West | Hamburg | refined | seed oil | conventional | no | large city |
| 23 | 4.89 | 1 | North-West | Hamburg | refined | seed oil | conventional | no | large city |
| 24 | 1.99 | 1 | North-West | Hamburg | refined | seed oil | conventional | no | large city |
| 25 | 3.12 | 1 | North-West | Hamburg | refined | seed oil | conventional | no | large city |
| 26 | 7.89 | 1 | North-West | Hamburg | refined | seed oil | conventional | no | large city |
| 27 | 3.59 | 1 | North-West | Hamburg | refined | seed oil | conventional | no | large city |
| 28 | 3.07 | 1 | North-West | Hamburg | cold pressed | kernel oil | conventional | no | large city |
| 29 | 0.79 | 1 | North-West | Hamburg | cold pressed | kernel oil | conventional | no | large city |
| 30 | 4.14 | 1 | North-West | Hamburg | refined | seed oil | conventional | no | large city |
| 31 | 3.10 | 1 | North-West | Hamburg | refined | seed oil | conventional | no | large city |
| 32 | 0.68 | 1 | North-West | Hamburg | refined | seed oil | conventional | no | large city |
| 33 | 2.57 | 1 | North-West | Hamburg | cold pressed | kernel oil | conventional | no | large city |
| 34 | 1.27 | 1 | North-West | Hamburg | refined | seed oil | conventional | no | large city |
| 35 | 0.63 | 1 | North-West | Hamburg | cold pressed | kernel oil | organic | no | large city |
| 37 | 4.09 | 1 | North-West | Hamburg | refined | seed oil | conventional | no | large city |
| 38 | 0.54 | 1 | North-West | Hamburg | refined | seed oil | conventional | no | large city |
| 39 | 2.50 | 1 | North-West | Hamburg | refined | seed oil | conventional | no | large city |
| 40 | 9.10 | 1 | East | Crivitz | refined | seed oil | conventional | no | rural area |
| 41 | 3.59 | 1 | East | Crivitz | refined | seed oil | conventional | no | rural area |
| 42 | 2.55 | 1 | East | Crivitz | refined | seed oil | conventional | no | rural area |
| 43 | 1.52 | 1 | East | Crivitz | cold pressed | kernel oil | conventional | no | rural area |
| 44 | 2.15 | 1 | East | Crivitz | refined | seed oil | conventional | no | rural area |
| 45 | 5.05 | 1 | East | Crivitz | cold pressed | seed oil | conventional | no | rural area |
| 46 | 4.05 | 1 | East | Crivitz | refined | seed oil | conventional | no | rural area |
| 47 | 2.61 | 1 | East | Berlin | cold pressed | seed oil | organic | no | large city |
| 48 | 1.39 | 1 | East | Berlin | cold pressed | kernel oil | conventional | no | large city |
| 49 | 2.33 | 1 | East | Berlin | refined | seed oil | conventional | no | large city |
| 50 | 0.50 | 1 | East | Berlin | refined | seed oil | conventional | no | large city |
| 51 | 0.90 | 1 | East | Berlin | cold pressed | kernel oil | organic | no | large city |
| 52 | 0.40 | 1 | East | Berlin | refined | seed oil | conventional | no | large city |
| 53 | 4.06 | 1 | East | Berlin | refined | seed oil | conventional | no | large city |
| 54 | 1.42 | 1 | East | Berlin | cold pressed | seed oil | organic | no | large city |
| 55 | 5.93 | 1 | East | Berlin | refined | seed oil | conventional | no | large city |
| 56 | 1.36 | 1 | East | Berlin | cold pressed | seed oil | conventional | no | large city |
| 57 | 6.21 | 1 | East | Berlin | refined | seed oil | conventional | no | large city |
| 58 | 1.24 | 1 | East | Berlin | cold pressed | seed oil | organic | no | large city |
| 59 | 0.87 | 1 | East | Berlin | cold pressed | kernel oil | conventional | no | large city |
| 60 | 2.02 | 1 | East | Berlin | refined | seed oil | conventional | no | large city |
| 61 | 0.93 | 1 | East | Berlin | refined | seed oil | conventional | no | large city |
| 62 | 0.37 | 1 | East | Berlin | refined | seed oil | organic | yes | large city |
| 63 | 1.92 | 1 | East | Berlin | refined | seed oil | conventional | no | large city |
| 64 | 4.07 | 1 | East | Berlin | cold pressed | seed oil | conventional | no | large city |
| 65 | 0.41 | 1 | East | Gera | refined | seed oil | conventional | no | medium-sized city |
| 66 | 2.80 | 1 | East | Gera | refined | seed oil | conventional | no | medium-sized city |
| 67 | 1.76 | 1 | East | Gera | refined | seed oil | conventional | no | medium-sized city |
| 68 | 1.74 | 1 | East | Gera | refined | seed oil | conventional | no | medium-sized city |
| 69 | 1.88 | 1 | East | Gera | refined | seed oil | conventional | no | medium-sized city |
| 70 | 2.79 | 1 | East | Gera | cold pressed | seed oil | organic | no | medium-sized city |
| 71 | 3.25 | 1 | East | Gera | refined | seed oil | conventional | no | medium-sized city |
| 72 | 5.07 | 1 | East | Gera | refined | seed oil | conventional | no | medium-sized city |
| 73 | 0.45 | 1 | East | Gera | refined | seed oil | conventional | no | medium-sized city |
| 74 | 3.39 | 1 | East | Gera | refined | seed oil | conventional | no | medium-sized city |
| 75 | 1.38 | 1 | East | Gera | cold pressed | kernel oil | conventional | no | medium-sized city |
| 76 | 1.73 | 1 | East | Gera | refined | seed oil | conventional | no | medium-sized city |
| 77 | 1.88 | 1 | East | Gera | refined | seed oil | conventional | no | medium-sized city |
| 78 | 1.73 | 1 | West | Köln | refined | seed oil | conventional | no | large city |
| 79 | 4.92 | 1 | West | Köln | refined | seed oil | conventional | no | large city |
| 80 | 0.93 | 1 | West | Köln | cold pressed | seed oil | organic | no | large city |
| 81 | 2.29 | 1 | West | Köln | refined | seed oil | conventional | no | large city |
| 82 | 3.26 | 1 | West | Köln | refined | seed oil | conventional | no | large city |
| 83 | 7.29 | 1 | West | Köln | cold pressed | kernel oil | conventional | no | large city |
| 84 | 0.63 | 1 | West | Köln | cold pressed | kernel oil | organic | no | large city |
| 85 | 0.44 | 1 | West | Köln | refined | seed oil | conventional | no | large city |
| 86 | 3.33 | 1 | West | Köln | refined | seed oil | conventional | no | large city |
| 87 | 0.54 | 1 | West | Köln | refined | seed oil | conventional | no | large city |
| 88 | 3.64 | 1 | West | Köln | cold pressed | kernel oil | conventional | no | large city |
| 89 | 5.96 | 1 | West | Köln | refined | seed oil | conventional | no | large city |
| 90 | 3.86 | 1 | West | Köln | cold pressed | seed oil | conventional | no | large city |
| 91 | 1.45 | 1 | West | Köln | cold pressed | seed oil | organic | no | large city |
| 92 | 4.15 | 1 | West | Köln | refined | seed oil | conventional | no | large city |
| 93 | 4.40 | 1 | West | Köln | refined | seed oil | conventional | no | large city |
| 94 | 2.83 | 1 | West | Worms | refined | seed oil | conventional | no | medium-sized city |
| 95 | 3.35 | 1 | West | Kirtorf | refined | seed oil | conventional | no | rural area |
| 96 | 0.62 | 1 | West | Kirtorf | cold pressed | kernel oil | organic | no | rural area |
| 97 | 6.16 | 1 | West | Kirtorf | refined | seed oil | conventional | no | rural area |
| 98 | 3.45 | 1 | West | Kirtorf | refined | seed oil | conventional | no | rural area |
| 99 | 7.06 | 1 | West | Kirtorf | refined | seed oil | conventional | no | rural area |
| 100 | 2.07 | 1 | West | Kirtorf | refined | seed oil | conventional | no | rural area |
| 101 | 1.81 | 1 | South | Aalen | refined | seed oil | conventional | no | medium-sized city |
| 102 | 2.28 | 1 | South | Aalen | cold pressed | seed oil | conventional | no | medium-sized city |
| 103 | 0.89 | 1 | South | Aalen | cold pressed | kernel oil | conventional | no | medium-sized city |
| 104 | 7.11 | 1 | South | Aalen | refined | seed oil | conventional | no | medium-sized city |
| 105 | 0.80 | 1 | South | Aalen | cold pressed | seed oil | organic | no | medium-sized city |
| 106 | 5.19 | 1 | South | Aalen | refined | seed oil | conventional | no | medium-sized city |
| 107 | 3.04 | 1 | South | Aalen | cold pressed | kernel oil | conventional | no | medium-sized city |
| 108 | 0.49 | 1 | South | Aalen | refined | seed oil | conventional | no | medium-sized city |
| 109 | 5.99 | 1 | South | Aalen | refined | seed oil | conventional | no | medium-sized city |
| 110 | 1.77 | 1 | South | Aalen | cold pressed | seed oil | conventional | no | medium-sized city |
| 111 | 2.43 | 1 | South | Aalen | refined | seed oil | conventional | no | medium-sized city |
| 112 | 3.14 | 1 | South | Aalen | refined | seed oil | conventional | no | medium-sized city |
| 113 | 1.23 | 1 | South | Aalen | cold pressed | seed oil | organic | no | medium-sized city |
| 114 | 5.98 | 1 | South | München | refined | seed oil | conventional | no | large city |
| 115 | 0.77 | 1 | South | München | refined | kernel oil | conventional | no | large city |
| 116 | 3.53 | 1 | South | München | refined | seed oil | conventional | no | large city |
| 117 | 0.48 | 1 | South | München | refined | seed oil | conventional | no | large city |
| 118 | 5.04 | 1 | South | München | refined | seed oil | conventional | no | large city |
| 119 | 4.83 | 1 | South | München | refined | seed oil | conventional | no | large city |
| 120 | 1.79 | 1 | South | München | refined | seed oil | conventional | no | large city |
| 121 | 1.72 | 1 | South | München | refined | seed oil | conventional | no | large city |
| 122 | 5.82 | 1 | South | München | cold pressed | seed oil | conventional | no | large city |
| 123 | 3.00 | 1 | South | München | refined | seed oil | conventional | no | large city |
| 124 | 1.80 | 1 | South | München | refined | seed oil | conventional | no | large city |
| 125 | 1.58 | 1 | South | München | refined | seed oil | conventional | no | large city |
| 126 | 2.47 | 1 | South | München | refined | seed oil | conventional | no | large city |
| 127 | 0.39 | 1 | South | München | refined | seed oil | conventional | no | large city |
| 128 | 0.48 | 1 | South | München | cold pressed | seed oil | conventional | no | large city |
| 129 | 2.78 | 1 | South | München | refined | seed oil | conventional | no | large city |
| 130 | 0.50 | 1 | South | München | cold pressed | seed oil | organic | no | large city |
| 131 | 3.40 | 1 | South | Nittenau | refined | seed oil | conventional | no | rural area |
| 132 | 5.53 | 1 | South | Nittenau | refined | seed oil | conventional | no | rural area |
| 133 | 0.78 | 1 | South | Nittenau | cold pressed | seed oil | organic | no | rural area |
| 134 | 6.86 | 1 | South | Nittenau | refined | seed oil | conventional | no | rural area |
| 135 | 0.73 | 1 | South | Nittenau | cold pressed | kernel oil | conventional | no | rural area |
| 136 | 3.44 | 1 | South | Nittenau | refined | seed oil | conventional | no | rural area |
| 137 | 1.96 | 1 | South | Nittenau | refined | seed oil | conventional | no | rural area |
| 138 | 3.59 | 1 | West | Worms | cold pressed | kernel oil | conventional | no | medium-sized city |
| 139 | 1.53 | 1 | West | Worms | cold pressed | kernel oil | conventional | no | medium-sized city |
| 140 | 4.62 | 1 | West | Worms | refined | seed oil | conventional | no | medium-sized city |
| 141 | 0.57 | 1 | West | Worms | refined | seed oil | conventional | no | medium-sized city |
| 142 | 3.18 | 1 | West | Worms | refined | seed oil | conventional | no | medium-sized city |
| 143 | 4.53 | 1 | West | Worms | cold pressed | kernel oil | conventional | no | medium-sized city |
| 144 | 1.70 | 1 | West | Worms | refined | seed oil | conventional | no | medium-sized city |
| 145 | 2.89 | 1 | West | Worms | refined | seed oil | conventional | no | medium-sized city |
| 146 | 0.44 | 1 | West | Worms | refined | seed oil | conventional | no | medium-sized city |
| 147 | 0.76 | 1 | West | Worms | refined | seed oil | conventional | no | medium-sized city |
| 148 | 0.96 | 1 | West | Worms | refined | seed oil | organic | yes | medium-sized city |
| 149 | 2.84 | 1 | West | Worms | refined | seed oil | conventional | no | medium-sized city |
| 150 | 1.45 | 1 | West | Worms | refined | seed oil | conventional | no | medium-sized city |
| 151 | 0.52 | 1 | West | Worms | cold pressed | seed oil | conventional | no | medium-sized city |
| 158 | 4.08 | 2 | South | Nittenau | refined | seed oil | conventional | no | rural area |
| 159 | 1.82 | 2 | South | Nittenau | cold pressed | kernel oil | conventional | no | rural area |
| 160 | 3.44 | 2 | South | Nittenau | refined | seed oil | conventional | no | rural area |
| 161 | 4.51 | 2 | South | Nittenau | cold pressed | kernel oil | conventional | no | rural area |
| 162 | 1.06 | 2 | South | Nittenau | cold pressed | kernel oil | conventional | no | rural area |
| 163 | 4.90 | 2 | South | Nittenau | refined | seed oil | conventional | no | rural area |
| 164 | 4.69 | 2 | South | Nittenau | refined | seed oil | conventional | no | rural area |
| 165 | 4.51 | 2 | South | Nittenau | refined | seed oil | conventional | no | rural area |
| 166 | 2.86 | 2 | South | München | refined | seed oil | conventional | no | large city |
| 167 | 1.33 | 2 | South | München | cold pressed | seed oil | organic | no | large city |
| 168 | 1.15 | 2 | South | München | refined | seed oil | conventional | no | large city |
| 169 | 0.31 | 2 | South | München | cold pressed | seed oil | conventional | no | large city |
| 170 | 2.50 | 2 | South | München | refined | seed oil | conventional | no | large city |
| 171 | 0.77 | 2 | South | München | cold pressed | seed oil | organic | no | large city |
| 172 | 0.98 | 2 | South | München | cold pressed | kernel oil | organic | no | large city |
| 173 | 0.67 | 2 | South | München | refined | seed oil | conventional | no | large city |
| 174 | 0.75 | 2 | South | München | cold pressed | kernel oil | conventional | no | large city |
| 175 | 2.65 | 2 | South | München | refined | seed oil | conventional | no | large city |
| 176 | 3.02 | 2 | South | München | refined | seed oil | conventional | no | large city |
| 177 | 0.47 | 2 | South | München | refined | seed oil | conventional | no | large city |
| 178 | 1.64 | 2 | South | München | cold pressed | seed oil | organic | no | large city |
| 179 | 2.56 | 2 | South | München | refined | seed oil | conventional | no | large city |
| 180 | 3.18 | 2 | South | München | refined | seed oil | conventional | no | large city |
| 181 | 4.82 | 2 | South | München | refined | seed oil | conventional | no | large city |
| 182 | 1.51 | 2 | South | München | cold pressed | seed oil | organic | no | large city |
| 183 | 1.92 | 2 | South | München | refined | seed oil | conventional | no | large city |
| 184 | 3.23 | 2 | South | Aalen | refined | seed oil | conventional | no | medium-sized city |
| 185 | 3.85 | 2 | South | Aalen | refined | seed oil | conventional | no | medium-sized city |
| 186 | 5.67 | 2 | South | Aalen | refined | seed oil | conventional | no | medium-sized city |
| 187 | 1.19 | 2 | South | Aalen | refined | seed oil | organic | yes | medium-sized city |
| 188 | 0.67 | 2 | South | Aalen | cold pressed | kernel oil | organic | no | medium-sized city |
| 189 | 3.81 | 2 | South | Aalen | cold pressed | seed oil | conventional | no | medium-sized city |
| 190 | 2.12 | 2 | South | Aalen | refined | seed oil | conventional | no | medium-sized city |
| 191 | 0.67 | 2 | South | Aalen | refined | seed oil | conventional | no | medium-sized city |
| 192 | 3.21 | 2 | South | Aalen | refined | seed oil | conventional | no | medium-sized city |
| 193 | 3.07 | 2 | South | Aalen | refined | seed oil | conventional | no | medium-sized city |
| 194 | 2.86 | 2 | South | Aalen | refined | seed oil | conventional | no | medium-sized city |
| 195 | 0.52 | 2 | South | Aalen | refined | seed oil | conventional | no | medium-sized city |
| 196 | 1.18 | 2 | West | Worms | cold pressed | seed oil | conventional | no | medium-sized city |
| 197 | 3.19 | 2 | West | Worms | refined | seed oil | conventional | no | medium-sized city |
| 198 | 2.49 | 2 | West | Worms | refined | seed oil | conventional | no | medium-sized city |
| 199 | 3.49 | 2 | West | Worms | refined | seed oil | conventional | no | medium-sized city |
| 200 | 2.36 | 2 | West | Worms | refined | seed oil | conventional | no | medium-sized city |
| 201 | 4.07 | 2 | West | Worms | cold pressed | seed oil | conventional | no | medium-sized city |
| 202 | 2.69 | 2 | West | Worms | refined | seed oil | conventional | no | medium-sized city |
| 203 | 2.78 | 2 | West | Worms | refined | seed oil | conventional | no | medium-sized city |
| 204 | 3.23 | 2 | West | Worms | cold pressed | seed oil | organic | no | medium-sized city |
| 205 | 3.14 | 2 | West | Worms | refined | seed oil | conventional | no | medium-sized city |
| 206 | 2.70 | 2 | West | Worms | refined | seed oil | conventional | no | medium-sized city |
| 207 | 1.09 | 2 | West | Worms | cold pressed | kernel oil | conventional | no | medium-sized city |
| 208 | 2.64 | 2 | West | Kirtorf | refined | seed oil | conventional | no | rural area |
| 209 | 2.64 | 2 | West | Kirtorf | refined | seed oil | conventional | no | rural area |
| 210 | 3.65 | 2 | West | Kirtorf | cold pressed | seed oil | organic | no | rural area |
| 211 | 3.03 | 2 | West | Kirtorf | refined | seed oil | conventional | no | rural area |
| 212 | 0.46 | 2 | West | Kirtorf | refined | seed oil | conventional | no | rural area |
| 213 | 3.31 | 2 | West | Kirtorf | refined | seed oil | conventional | no | rural area |
| 214 | 7.30 | 2 | West | Kirtorf | refined | seed oil | conventional | no | rural area |
| 215 | 2.46 | 2 | West | Köln | refined | seed oil | conventional | no | large city |
| 216 | 2.80 | 2 | West | Köln | cold pressed | kernel oil | conventional | no | large city |
| 217 | 0.50 | 2 | West | Köln | refined | seed oil | conventional | no | large city |
| 218 | 2.56 | 2 | West | Köln | refined | seed oil | conventional | no | large city |
| 219 | 2.46 | 2 | West | Köln | refined | seed oil | conventional | no | large city |
| 220 | 2.04 | 2 | West | Köln | cold pressed | kernel oil | conventional | no | large city |
| 221 | 0.69 | 2 | West | Köln | cold pressed | kernel oil | conventional | no | large city |
| 222 | 4.01 | 2 | West | Köln | refined | seed oil | conventional | no | large city |
| 223 | 2.33 | 2 | West | Köln | refined | seed oil | conventional | no | large city |
| 224 | 0.85 | 2 | West | Köln | refined | seed oil | conventional | no | large city |
| 225 | 7.23 | 2 | West | Köln | refined | seed oil | conventional | no | large city |
| 226 | 0.43 | 2 | West | Köln | refined | seed oil | conventional | no | large city |
| 227 | 4.18 | 2 | West | Köln | cold pressed | kernel oil | conventional | no | large city |
| 228 | 3.11 | 2 | West | Köln | refined | seed oil | conventional | no | large city |
| 229 | 0.75 | 2 | West | Köln | cold pressed | seed oil | organic | no | large city |
| 230 | 3.27 | 2 | West | Köln | cold pressed | kernel oil | conventional | no | large city |
| 231 | 3.48 | 2 | West | Köln | refined | seed oil | conventional | no | large city |
| 232 | 3.40 | 2 | West | Köln | refined | seed oil | conventional | no | large city |
| 233 | 7.86 | 2 | West | Köln | cold pressed | seed oil | conventional | no | large city |
| 234 | 2.67 | 2 | East | Gera | refined | seed oil | conventional | no | medium-sized city |
| 235 | 3.42 | 2 | East | Gera | refined | seed oil | conventional | no | medium-sized city |
| 236 | 1.78 | 2 | East | Gera | cold pressed | seed oil | conventional | no | medium-sized city |
| 237 | 0.44 | 2 | East | Gera | refined | seed oil | conventional | no | medium-sized city |
| 238 | 2.63 | 2 | East | Gera | refined | seed oil | conventional | no | medium-sized city |
| 239 | 0.17 | 2 | East | Gera | refined | seed oil | conventional | no | medium-sized city |
| 240 | 2.83 | 2 | East | Gera | refined | seed oil | conventional | no | medium-sized city |
| 241 | 3.86 | 2 | East | Gera | cold pressed | seed oil | conventional | no | medium-sized city |
| 242 | 2.73 | 2 | East | Gera | refined | seed oil | conventional | no | medium-sized city |
| 243 | 3.90 | 2 | East | Gera | cold pressed | kernel oil | conventional | no | medium-sized city |
| 244 | 0.24 | 2 | East | Gera | refined | seed oil | conventional | no | medium-sized city |
| 245 | 0.45 | 2 | East | Gera | refined | seed oil | conventional | no | medium-sized city |
| 246 | 9.68 | 2 | East | Berlin | refined | seed oil | conventional | no | large city |
| 247 | 0.20 | 2 | East | Berlin | refined | seed oil | conventional | no | large city |
| 248 | 2.78 | 2 | East | Berlin | refined | seed oil | conventional | no | large city |
| 249 | 0.73 | 2 | East | Berlin | cold pressed | seed oil | organic | no | large city |
| 250 | 0.97 | 2 | East | Berlin | cold pressed | kernel oil | conventional | no | large city |
| 251 | 3.09 | 2 | East | Berlin | refined | seed oil | conventional | no | large city |
| 252 | 0.45 | 2 | East | Berlin | refined | seed oil | conventional | no | large city |
| 253 | 3.10 | 2 | East | Berlin | refined | seed oil | conventional | no | large city |
| 254 | 3.24 | 2 | East | Berlin | refined | seed oil | conventional | no | large city |
| 255 | 2.05 | 2 | East | Berlin | refined | seed oil | conventional | no | large city |
| 256 | 1.81 | 2 | East | Berlin | refined | seed oil | conventional | no | large city |
| 257 | 1.08 | 2 | East | Berlin | cold pressed | kernel oil | conventional | no | large city |
| 258 | 3.63 | 2 | East | Berlin | refined | seed oil | conventional | no | large city |
| 259 | 1.65 | 2 | East | Berlin | cold pressed | kernel oil | conventional | no | large city |
| 260 | 4.16 | 2 | East | Berlin | refined | seed oil | conventional | no | large city |
| 261 | 0.45 | 2 | East | Berlin | refined | seed oil | conventional | no | large city |
| 262 | 2.58 | 2 | East | Berlin | cold pressed | kernel oil | conventional | no | large city |
| 263 | 3.02 | 2 | East | Crivitz | refined | seed oil | conventional | no | rural area |
| 264 | 4.19 | 2 | East | Crivitz | refined | seed oil | conventional | no | rural area |
| 265 | 2.41 | 2 | East | Crivitz | refined | seed oil | conventional | no | rural area |
| 266 | 5.11 | 2 | East | Crivitz | cold pressed | seed oil | conventional | no | rural area |
| 267 | 2.42 | 2 | East | Crivitz | refined | seed oil | conventional | no | rural area |
| 268 | 2.98 | 2 | East | Crivitz | refined | seed oil | conventional | no | rural area |
| 269 | 4.10 | 2 | East | Crivitz | cold pressed | kernel oil | conventional | no | rural area |
| 270 | 2.67 | 2 | East | Crivitz | refined | seed oil | conventional | no | rural area |
| 271 | 3.46 | 2 | North-West | Hamburg | refined | seed oil | conventional | no | large city |
| 272 | 0.55 | 2 | North-West | Hamburg | refined | seed oil | conventional | no | large city |
| 273 | 1.89 | 2 | North-West | Hamburg | refined | seed oil | conventional | no | large city |
| 274 | 2.85 | 2 | North-West | Hamburg | refined | seed oil | conventional | no | large city |
| 275 | 3.77 | 2 | North-West | Hamburg | refined | seed oil | conventional | no | large city |
| 276 | 2.41 | 2 | North-West | Hamburg | refined | seed oil | conventional | no | large city |
| 277 | 0.41 | 2 | North-West | Hamburg | refined | seed oil | conventional | no | large city |
| 278 | 6.35 | 2 | North-West | Hamburg | refined | seed oil | conventional | no | large city |
| 279 | 3.06 | 2 | North-West | Hamburg | cold pressed | seed oil | organic | no | large city |
| 280 | 2.98 | 2 | North-West | Celle | cold pressed | kernel oil | organic | no | medium-sized city |
| 281 | 8.77 | 2 | North-West | Hamburg | cold pressed | seed oil | conventional | no | large city |
| 282 | 2.89 | 2 | North-West | Hamburg | refined | seed oil | conventional | no | large city |
| 283 | 3.59 | 2 | North-West | Hamburg | refined | seed oil | conventional | no | large city |
| 284 | 6.40 | 2 | North-West | Hamburg | refined | seed oil | conventional | no | large city |
| 285 | 1.12 | 2 | North-West | Hamburg | refined | seed oil | organic | yes | large city |
| 286 | 3.46 | 2 | North-West | Hamburg | refined | seed oil | conventional | no | large city |
| 287 | 0.41 | 2 | North-West | Hamburg | refined | seed oil | conventional | no | large city |
| 288 | 3.22 | 2 | North-West | Celle | refined | seed oil | conventional | no | medium-sized city |
| 289 | 4.75 | 2 | North-West | Celle | refined | seed oil | conventional | no | medium-sized city |
| 290 | 4.12 | 2 | North-West | Celle | cold pressed | kernel oil | conventional | no | medium-sized city |
| 291 | 2.11 | 2 | North-West | Celle | refined | seed oil | conventional | no | medium-sized city |
| 292 | 2.78 | 2 | North-West | Celle | cold pressed | kernel oil | conventional | no | medium-sized city |
| 293 | 2.32 | 2 | North-West | Celle | cold pressed | seed oil | conventional | no | medium-sized city |
| 294 | 2.97 | 2 | North-West | Celle | refined | seed oil | conventional | no | medium-sized city |
| 295 | 0.80 | 2 | North-West | Celle | cold pressed | seed oil | organic | no | medium-sized city |
| 296 | 1.75 | 2 | North-West | Celle | refined | seed oil | conventional | no | medium-sized city |
| 297 | 2.97 | 2 | North-West | Celle | refined | seed oil | conventional | no | medium-sized city |
| 298 | 3.45 | 2 | North-West | Celle | refined | seed oil | conventional | no | medium-sized city |
| 299 | 3.00 | 2 | North-West | Celle | refined | seed oil | conventional | no | medium-sized city |
| 300 | 3.43 | 2 | North-West | Celle | refined | seed oil | conventional | no | medium-sized city |
| 301 | 5.37 | 2 | North-West | Augustfehn | refined | seed oil | conventional | no | rural area |
| 302 | 6.05 | 2 | North-West | Augustfehn | cold pressed | kernel oil | conventional | no | rural area |
| 303 | 2.57 | 2 | North-West | Augustfehn | refined | seed oil | conventional | no | rural area |
| 304 | 0.17 | 2 | North-West | Augustfehn | refined | seed oil | conventional | no | rural area |
| 305 | 2.95 | 2 | North-West | Augustfehn | cold pressed | kernel oil | conventional | no | rural area |
| 306 | 1.62 | 2 | North-West | Augustfehn | cold pressed | seed oil | conventional | no | rural area |
| 307 | 0.84 | 2 | North-West | Celle | cold pressed | seed oil | conventional | no | medium-sized city |

^1^ Samples that were not pure rapeseed oils, but mixtures with other vegetable oils, were excluded from the analysis. Thus, some sample IDs are missing in this table.
^2^ Sampling period 1: 10 April – 06 May, 2019; Sampling period 2: 27 June – 20 July, 2019
^3^ At the places in the rural areas, some samples were also bought in neighboring small towns or villages.
^4^ Specifically designated for the preparation of complementary foods for infants.
